# 伴IRF4重排的大B细胞淋巴瘤6例报告并文献复习

**DOI:** 10.3760/cma.j.issn.0253-2727.2022.06.006

**Published:** 2022-06

**Authors:** 颖 于, 琦 孙, 承文 李, 玉娇 贾, 薇 刘, 婷玉 王, 瑞 吕, 禹廷 阎, 刚 安, 录贵 邱, 德慧 邹, 树华 易

**Affiliations:** 中国医学科学院血液病医院（中国医学科学院血液学研究所），北京协和医学院，实验血液学国家重点实验室，国家血液系统疾病临床医学研究中心，细胞生态海河实验室，天津 300020 State Key Laboratory of Experimental Hematology, National Clinical Research Center for Blood Diseases, Haihe Laboratory of Cell Ecosystem, Institute of Hematology & Blood Diseases Hospital, Chinese Academy of Medical Sciences & Peking Union Medical College, Tianjin 300020, China

**Keywords:** B细胞淋巴瘤, 基因，IRF4, 病理学, 荧光原位杂交, 二代基因测序, B-cell lymphoma, Gene, IRF4, Pathology, Fluorescence in situ immunohybridization, Next-generation sequencing

## Abstract

**目的:**

探讨伴1RF4重排的大B细胞淋巴瘤的临床、病理学、遗传学特征。

**方法:**

收集中国医学科学院血液病医院淋巴瘤诊疗中心2017年12月至2021年10月收治的6例伴1RF4重排的大B细胞淋巴瘤患者的临床资料，包括病理学、荧光原位杂交、二代基因测序检测，并复习相关文献。

**结果:**

①6例患者中男3例，女3例，中位发病年龄33岁。6例患者中3例起病部位为扁桃体，2例为淋巴结，1例为背部肿物。6例患者均采取了RCDOP（利妥昔单抗、环磷酰胺、脂质体阿霉素、长春新碱、泼尼松）方案治疗，随访至2021年11月，6例患者全部存活。②形态学：5例为弥漫性生长，1例为滤泡性生长，瘤细胞体积均为中等至大，病灶多呈膨胀性生长，5例核分裂象易见，6例均未见“星空现象”。③免疫组化：4例为生发中心B细胞（GCB）型，2例为non-GCB型；6例均为CD20、PAX5、MUM1、BCL6阳性，CD5阴性；4例CD10阴性；3例BCL2阳性，2例c-MYC阳性。④荧光原位杂交：6例患者IRF4分离探针检测均呈阳性；5例进行BCL6基因重排检测，2例重排阳性；各有5例进行了BCL2、MYC基因重排检测，均为阴性。⑤二代测序：3例患者应用石蜡组织标本进行了二代测序，可见IRF4、TP53、IGLL5、MYD88等淋巴瘤相关基因突变。

**结论:**

伴IRF4重排的大B细胞淋巴瘤是一种具有独特临床、病理学、遗传学特征的罕见的大B细胞淋巴瘤。对该类型淋巴瘤的发病机制、治疗选择、长期预后仍需进一步探索。

## Abstract (en)

**Objective:**

To study the clinical, histopathological, and genetic features of large B-cell lymphoma (LBCL) with IRF4 rearrangement.

**Methods:**

Six patients presenting at our center between December 2017 and October 2021 were evaluated by pathological examination, fluorescence in situ hybridization, and next-generation sequencing. The relevant literature was reviewed.

**Results:**

①The study sample included three males and three females with a median age of 33 years. Three tumors were in the tonsils, two in the lymphoid nodes, and one in the dorsal lump. All patients were treated using the RCDOP (rituximab, cyclophosphamide, liposomal doxorubicin, vincristine, prednisone) regimen. All of them were alive at the time of follow-up in November 2021. ②Microscopic examination showed an entirely follicular pattern in one case and an entirely diffused pattern in 5 cases. The tumor cells were medium to large, and most of the lesions were dilatative with brisk mitotic activity (*n*＝five cases) and no starry sky pattern (*n*＝6 cases). ③Four cases exhibited a GCB phenotype, and the other two exhibited a non-GCB phenotype. All of the cases were positive for CD20, PAX-5, MUM, and BCL6, and negative for CD5. Moreover, CD10, BCL2, and c-MYC were positive in 4, 3, and 2 cases, respectively.④IRF4 gene rearrangement was identified in all cases, BCL6 gene rearrangement was detected in 5 cases, and 2 cases were positive. BCL2 and MYC gene rearrangement were performed in 5 cases, all negative. ⑤Three paraffin tissue samples were used for next-generation sequencing, and lymphoma-related gene mutations such as IRF4, TP53, IGLL5, and MYD88 were detected in 3 cases.

**Conclusion:**

LBCL with IRF4 rearrangement is a rare entity with unique clinical, pathological, and genetic characteristics. This entity's pathogenesis, treatment options, and long-term prognosis still need to be explored further.

干扰素调节因子4（interferon regulatory factor 4，IRF 4）是干扰素家族的转录因子成员之一，因最早在骨髓瘤疾病中进行定义，故又称其为多发性骨髓瘤癌基因1（multiple myeloma gene 1，MUM1）。IRF4可调节B细胞向浆细胞分化，因此较特异地表达于淋巴细胞[1]，同时在巨噬细胞、树突状细胞、髓系细胞、浆细胞也同样高表达[2]。

伴IRF4重排的大B细胞淋巴瘤（large B-cell lymphoma）在2016版WHO淋巴瘤分类中，正式作为一个暂定的亚类被命名[3]。该类型十分罕见，主要发生在儿童及青少年，占成熟B细胞淋巴瘤的5％～6％[4]–[5]。当前对该类型淋巴瘤研究报道较少，认识不足，本研究报道6例伴IRF4重排的大B细胞淋巴瘤患者的临床、病理学、遗传学特征及预后。

## 病例与方法

1. 病例：纳入中国医学科学院血液病医院淋巴瘤诊疗中心2017年12月至2021年10月确诊的伴IRF4重排的大B细胞淋巴瘤6例。收集患者临床特征、病理检查、血液学相关实验室检查、治疗方案等相关信息。患者病理结果均通过2名以上三甲医院病理科医师复核。并通过电话对患者进行随访。

2. 免疫组化：对这6例患者的组织切片（4 µm）进行免疫组化染色。采用EnVision两步法，并设立阴性、阳性对照。使用特异性CD10、PAX5、CD21抗体（上海杰浩生物技术有限公司产品），CD20、CD79a、CD3、CD5、BCL2抗体（泉辉国际企业有限公司产品），CD19、BCL6、IRF4（MUM1）抗体（中杉金桥生物有限公司产品）。MUM1、BCL2、BCL6的阳性阈值分别为30％、50％、30％。

3. 荧光原位杂交（FISH）：IRF4基因重排检测使用双色分离探针（安必平医药科技股份有限公司产品），阳性阈值4.17％；5′IRF4（6p25）基因标记为红色（R），3′IRF4（6p25）基因标记为绿色（G），IRF4基因显示为黄色或红绿叠加信号（F），正常信号为2F，阳性信号为1F1R1G。TP53基因缺失探针（安必平医药科技股份有限公司产品）阳性阈值7.8％。MYC基因重排、BCL2基因重排、BCL6基因重排阳性阈值分别为3.57％、3.5％、2.42％（雅康博生物科技有限公司产品）。

4. 二代基因测序:使用TINamp Micro Kit试剂盒（天根生化科技有限公司产品）从石蜡组织切片中提取DNA。使用紫外线吸收分析器（THERMO ND 2000）进行DNA浓度检测。采用Illumina NextSeq 550测序平台，对125个与淋巴瘤密切相关的基因蛋白编码区域的点突变和短片段插入/缺失突变进行高通量测序，平均测序深度为2000×。本研究中对3例该类型淋巴瘤患者石蜡组织标本进行了二代测序检测。

## 结果

一、临床特征

6例患者中，男3例，女3例，中位发病年龄33（13～58）岁。6例患者中3例起病部位为扁桃体，2例为淋巴结，1例为背部肿物。4例分期为Ⅰ期，2例为Ⅱ期。6例患者均进行PET-CT检查，例1、2、3、6在完整切除扁桃体后行PET-CT未见异常；例4切除扁桃体后行PET-CT可见右颈部1.7 cm×1.4 cm肿大淋巴结，SUVmax 58；例5切除扁桃体后PET-CT提示左侧下牙槽骨区高代谢灶，SUVmax 9.6，颈部双侧1.4 cm×0.8 cm高代谢淋巴结，SUVmax 6.6。所有患者IPI、aaIPI评分均为0分。1例患者同时合并乙型肝炎病毒感染，其余患者均无其他合并疾病，所有患者均无家族史及遗传病史。6例患者均采取了RCDOP方案（利妥昔单抗、环磷酰胺、脂质体阿霉素、长春新碱、泼尼松）治疗，3例患者进行了疗效评价，全部为完全缓解（CR），随访至2021年11月，6例患者全部存活，进行了疗效评价的3例患者仍为持续CR状态。患者临床特征详见表1。

**表1:** 6例伴IRF4重排的大B细胞淋巴瘤患者临床特征

例号	性别	年龄（岁）	发病部位	肿物大小（cm）	分期	IPI评分	治疗方案	疗效评价	随访时间（月）	生存状态
1	男	13	耳前淋巴结	2.2×1.4	Ⅰ期	0分	RCDOP	CR	47	存活
2	男	14	颈部淋巴结	5×3	Ⅰ期	0分	RCDOP	CR	28	存活
3	女	40	背部肿物	2.2×1.1	Ⅰ期	0分	RCDOP	CR	4	存活
4	女	58	扁桃体	NA	Ⅱ期	0分	RCDOP	NA	1	存活
5	男	32	扁桃体	NA	Ⅱ期	0分	RCDOP	NA	1	存活
6	女	34	扁桃体	NA	Ⅰ期	0分	RCDOP	NA	1	存活

注：RCDOP：利多昔单抗+环磷酰胺+脂质体阿霉素+长春新碱+泼尼松；CR：完全缓解；NA：未获得

二、病理特征

6例病理标本均为完整切除所得。3例扁桃体样本均可见被覆鳞状上皮。例1可见肿瘤细胞呈滤泡样生长，其余5例标本均呈弥漫性生长。病灶多呈现膨胀性生长，表现为异形滤泡体积大，紧密排列，周围套区不完整或消失，其间见被挤压的残存淋巴组织；例4组织少部分区域可见肿瘤细胞结节状分布，结节内可见FDC网。需要注意的是，位于扁桃体的病变在浅表黏膜下的纤维间质中则表现为浸润性生长。高倍镜下，6例标本中肿瘤细胞胞体均中等至大，表现为体积偏大的中心母细胞样或核型不规则的母细胞样状态，染色质较粗，部分可见核仁，例2～6均易见核分裂象。6例标本均未见“星空现象”（图1）。

**图1:**
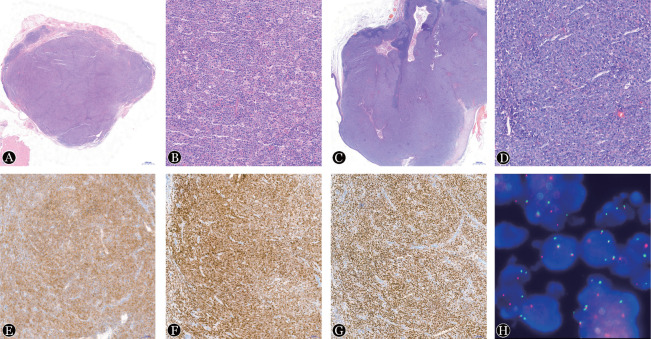
伴IRF4重排的大B细胞淋巴瘤患者病理及荧光原位杂交结果 A：例1病变呈滤泡型生长模式（HE染色，低倍）；B：例1生发中心内细胞成分以中心细胞和中心母细胞为主，滤泡间区可见肿瘤细胞浸润（HE染色，高倍）；C：伴有肿瘤呈弥漫性生长模式，淋巴结大部分肿瘤占据，剩余被挤压正常组织（HE染色，低倍）；D：肿瘤细胞形态较一致，染色质粗糙，可见核仁，核分裂象易见（HE染色，低倍）；E：肿瘤细胞CD20阳性（EnVision法，高倍）；F：肿瘤细胞MUM1阳性（EnVision法，高倍）；G：肿瘤细胞BCL6阳性（EnVision法，高倍）；H：例3 77％以上的细胞岀现1红1绿1黄信号，提示IRF4基因断裂重排（FISH，高倍）

免疫组化检测显示，6例标本CD5均为阴性，B细胞标志CD20、PAX5均为阳性；同时6例标本也均呈MUM1、BCL6阳性；4例呈CD10阳性，为生发中心B细胞（GCB）亚型；2例为CD10阴性，同时标本BCL6阳性，MUM1阳性，为non-GCB亚型；3例BCL2阳性；2例检测到c-MYC阳性（阳性率40％～50％）；5例标本Ki67指数较高，超过70％（70％～95％），例1标本肿瘤细胞呈滤泡性生长，Ki67指数偏低，为40％。4例标本进行了EBER杂交，均为阴性，详见表2。

**表2:** 6例伴IRF4重排的大B细胞淋巴瘤患者病理及荧光原位杂交（FISH）结果

例号	生长模式	细胞大小	免疫组化结果							FISH结果				
			MUM-1（％）	BCL6（％）	CD10（％）	BCL2（％）	MYC（％）	Ki67指数（％）	EBER杂交	IRF4重排	BCL6重排	BCL2重排	MYC重排	TP53缺失
1	滤泡性	大细胞	+	+	+	+	−（30）	40	NA	+	NA	NA	NA	NA
2	弥漫性	大细胞	+	+	+	−	+（40～50）	70～80	−	+	−	−	−	NA
3	弥漫性	大细胞	+（40）	+（60）	+	−	−（30）	70～80	NA	+	−	−	−	+
4	弥漫性	大细胞	+（>90）	+（>90）	+	+（50）	+（热点区域阳性率50）	95	−	+	+	−	−	−
5	弥漫性	大细胞	+（>80）	+（70）	−	−	−（10～20）	80～90	−	+	−	−	−	−
6	弥漫性	大细胞	+（80～90）	+（90）	−	+（80～90）	−（10）	80～90	−	+	+	−	−	−

注：NA：未检测

三、遗传学及分子生物学

1. FISH：6例患者进行了IRF4分离探针检测，均呈阳性；例1在肿瘤组织中不仅检测到1F1G1R的典型信号特征，同时可检测出1F1G、1R1G的不典型信号特征以及30％的细胞内可见1F的异常信号；例3同时可见多倍体阳性；例4部分细胞同样可以出现1F1G的不典型信号特征，阳性主要见于多倍体细胞。5例患者进行了BCL6基因重排检测，2例重排阳性；各有5例进行了BCL2、MYC基因重排检测，均为阴性；4例进行了TP53基因缺失检测，1例为阳性（表2）。例3在进行FISH检测时可见多倍体阳性及基因拷贝数改变，该患者的石蜡组织二代测序也提示了6、7、14号染色体部分区段可见拷贝数改变，但该患者的染色体核型检查未见异常。其余5例患者染色体核型均未见异常。

2. 二代测序：3例患者应用石蜡组织标本进行了二代测序。例3检出IRF4多位点突变，以及6、7、14号染色体部分区段可见拷贝数改变；例4检出KRAS、IDH2、PIM1、BTG2、FAT1突变，以及TP53、IGLL5多位点突变；例5除IRF4突变外，还可见IGLL5、FAT1、MYD88、DTX1、TNFAIP3、KMT2C突变。

## 讨论

2001年Tamura等[6]在探索IGH易位伙伴基因时通过双色FISH技术报道了第一例伴IRF4重排的大B细胞淋巴瘤。2011年Salaverria等[7]鉴定出14例具有IRF4重排的大B细胞淋巴瘤，同时发现这类淋巴瘤呈明显的惰性病程，预后良好。随着对该类型淋巴瘤报道的逐渐增多，越来越多的报道表明伴有IRF4重排的大B细胞淋巴瘤具有独特的生物学特征，并在2016版WHO造血与淋巴组织肿瘤分类中正式成为暂定的一个新亚类[3]。

MUM1/IRF4基因编码的转录因子IRF4，是IRF转录因子家族中的一种，编码MUM1蛋白。MUM1主要表达于浆细胞、活化的T细胞以及少部分生发中心“亮区”的中心细胞[2]。正常生发中心中，MUM1和BCL6几乎不同时表达[8]，而BCL6是维持正常生发中心形成的重要转录因子[9]，因此在GCB型弥漫大B细胞淋巴瘤免疫组化中通常MUM1阴性，BCL6阳性。伴IRF4重排的大B细胞淋巴瘤大部分为GCB型，但由于t（6;14）（p25;q32），即MUM1易位到第14号染色体IgH增强子位点，易位导致MUM1蛋白过表达，因而免疫组化中表现为MUM1、BCL6同时阳性。本文报道的6例患者免疫组化也均呈现MUM1和BCL6同时阳性。

根据当前的病例报道，伴IRF4重排的大B细胞淋巴瘤好发于儿童及青少年，肿瘤最常出现在韦氏环，头部、颈部以及胃肠道的淋巴结，通常为Ⅰ～Ⅱ期患者[4],[7],[10]–[11]。本文的6例患者中，例4为58岁，例3发病部位为背部肿物，均为不常见发病年龄和部位。Zhou等[12]报道了13例患者，显示该类型淋巴瘤发病年龄最大可至64岁，可累及唾液腺，同时该患者合并了艾滋病（HIV）。Chisholm等[4]也报道了1例发生在扁桃体，但起病时即为Ⅳ期（中枢侵犯）的特殊病例。不论是合并HIV或者分期晚，患者在接受系统治疗后均获得了CR，这2例患者分别随访17和36个月，均处于CR状态。提示该类型淋巴瘤群体仍具有异质性。起病晚期、发病部位不典型、分期晚、合并其他疾病是否提示更侵袭性的病程和更差的预后仍需更多的病例、更长的随访得出结论。

作为一个罕见疾病，当前对该疾病的治疗尚无统一的标准及共识。对该类型淋巴瘤的治疗仍然是参照弥漫大B细胞淋巴瘤的治疗。FLYER研究结果显示，对于年轻低危的侵袭性B细胞淋巴瘤和Ⅰ～Ⅱ期DLBCL患者，4个周期CHOP方案化疗均显示相同的结果[13]。该类型淋巴瘤好发于儿童及青少年，呈惰性病程，预后良好。对于病灶局限，已完整切除病灶，能否不再采取后续化疗，或者行短疗程局部放疗以减少化疗药物的不良反应仍需要探索。

在该类型淋巴瘤中，IRF4的重排主要是由IRF4基因断裂后进一步与IGH结构融合所致，少部分发生IGL/IRF4或IGK/IRF4的结构融合。部分患者存在BCL6基因重排，几乎所有患者都无MYC和BCL2基因重排。而当前无商品化的IRF4/IGH双色融合探针用于血液肿瘤检测，所以对IRF4重排的鉴定主要是依靠IRF4的分离探针进行检测。典型的IRF4重排显示为黄色或红绿叠加信号，正常信号特征为2F，阳性信号特征为1F1R1G。例1、例4在肿瘤组织中不仅检测到1F1G1R的典型信号特征，同时检出1F1G、1R1G的不典型信号特征以及例1 30％的细胞内可见1F的异常信号。出现不典型信号推测是IRF4与伙伴基因发生了不平衡易位以及断裂的位点位于红色基因标记处，也不排除标本制备过程中因为切片过薄导致的部分信号丢失。Salaverria等[7]也报道了3例伴非典型的IRF4重排的大B细胞淋巴瘤，国内也有伴不典型IRF4重排大B细胞淋巴瘤的病例报道[14]。这些病例提示其在临床特征、形态学、病理学以及分子突变等各方面与典型的IRF4重排相似，仍被认为是一种疾病。

当前对该疾病的发病机制仍不明确，研究者试图从分子学机制上进一步探索该类型淋巴瘤呈明显的惰性病程，区别于其他类型弥漫大B细胞淋巴瘤的原因。最新的研究从靶向测序、基因拷贝数改变、基因表达谱三方面进行了分析，发现该类型淋巴瘤最常出现IRF4和NF-κB信号通路相关基因（CARD11、CD79B、MYD88）突变，17p13.2片段缺失以及NF-κB信号通路下游基因的过表达[8],[15]。该通路的激活可能也与IRF4过表达有关[16]。同时CARD11、MAP2K1基因突变只分别出现在弥漫性、滤泡性生长的患者中，推测这些潜在的基因突变可能会影响肿瘤的形态学特征。根据当前所有报道的二代测序结果，除IRF4、CARD11、CD79B、MYD88、TP53外，还可见BTG2、IGLL5、PIM1基因突变[8],[12]，而这些突变均可在本文患者中发现。本文中的例3，FISH可见TP53基因的缺失，例4二代测序可见TP53基因的突变。需要注意的是，TP53作为一个抑癌基因，缺失或突变都提示疾病的不良预后，但在该类型淋巴瘤中，存在TP53的突变和缺失仍不能改变其惰性病程，似乎并不是不良的预后因素。而TP53基因在该类型淋巴瘤中预后价值仍需要更长时间的随访得出结论。同样需要注意的是二代测序检测到的IRF4基因突变并不能取代FISH的检测结果，诊断该类型淋巴瘤仍需依据IRF4分离探针结果。

综上所述，伴IRF4重排的大B细胞淋巴瘤是一种罕见的淋巴瘤类型，预后较好。在临床工作中遇到年轻、局限性、惰性病程、免疫组化提示CD10、MUM1、BCL6呈阳性的弥漫大B细胞淋巴瘤患者，可进一步加做IRF4分离探针检测协助进一步诊断与鉴别诊断。
